# Modeled Impacts of Chronic Wasting Disease on White-Tailed Deer in a Semi-Arid Environment

**DOI:** 10.1371/journal.pone.0163592

**Published:** 2016-10-06

**Authors:** Aaron M. Foley, David G. Hewitt, Charles A. DeYoung, Randy W. DeYoung, Matthew J. Schnupp

**Affiliations:** 1 Caesar Kleberg Wildlife Research Institute, Texas A&M University—Kingsville, Kingsville, Texas, United States of America; 2 King Ranch, Inc., Kingsville, Texas, United States of America; Colorado State University College of Veterinary Medicine and Biomedical Sciences, UNITED STATES

## Abstract

White-tailed deer are a culturally and economically important game species in North America, especially in South Texas. The recent discovery of chronic wasting disease (CWD) in captive deer facilities in Texas has increased concern about the potential emergence of CWD in free-ranging deer. The concern is exacerbated because much of the South Texas region is a semi-arid environment with variable rainfall, where precipitation is strongly correlated with fawn recruitment. Further, the marginally productive rangelands, in combination with erratic fawn recruitment, results in populations that are frequently density-independent, and thus sensitive to additive mortality. It is unknown how a deer population in semi-arid regions would respond to the presence of CWD. We used long-term empirical datasets from a lightly harvested (2% annual harvest) population in conjunction with 3 prevalence growth rates from CWD afflicted areas (0.26%, 0.83%, and 2.3% increases per year) via a multi-stage partially deterministic model to simulate a deer population for 25 years under four scenarios: 1) without CWD and without harvest, 2) with CWD and without harvest, 3) with CWD and male harvest only, and 4) with CWD and harvest of both sexes. The modeled populations without CWD and without harvest averaged a 1.43% annual increase over 25 years; incorporation of 2% annual harvest of both sexes resulted in a stable population. The model with slowest CWD prevalence rate growth (0.26% annually) without harvest resulted in stable populations but the addition of 1% harvest resulted in population declines. Further, the male age structure in CWD models became skewed to younger age classes. We incorporated fawn:doe ratios from three CWD afflicted areas in Wisconsin and Wyoming into the model with 0.26% annual increase in prevalence and populations did not begin to decline until ~10%, ~16%, and ~26% of deer were harvested annually. Deer populations in variable environments rely on high adult survivorship to buffer the low and erratic fawn recruitment rates. The increase in additive mortality rates for adults via CWD negatively impacted simulated population trends to the extent that hunter opportunity would be greatly reduced. Our results improve understanding of the potential influences of CWD on deer populations in semi-arid environments with implications for deer managers, disease ecologists, and policy makers.

## Introduction

Chronic-wasting disease (CWD), a transmissible spongiform encephalopathy, has had a large impact on the management of afflicted cervid populations in the United States and Canada [1–5]. Disease suppression efforts have proved difficult due to unwillingness of hunters to harvest deer as part of CWD-management strategies [6–8], time elapsed between disease emergence and detection [9], movements of free-ranging [10,11] and captive cervids [10], and persistence of prions in the environment [12–15]. Thus, where CWD is present, it is likely to remain endemic. In areas where CWD is not present, preventive measures will likely maximize efficacy of disease management efforts [1].

In June 2015, the first white-tailed deer (*Odocoileus virginanus*) in Texas tested positive for CWD [16]. To date, the disease has been found only in captive populations of white-tailed deer [17], yet the presence of CWD has major ramifications for management of all cervids in the region. Compared to other states and provinces afflicted with CWD, South Texas is unique in both its environment and in the cultural and economic importance of deer hunting. South Texas is a semi-arid environment with highly variable annual rainfall (CV >30%) [18]. The region is broadly characterized as having marginally productive vegetation communities [19], where rainfall greatly influences forage quality and quantity. During dry years, forage quality limits the physical ability of females to recruit fawns because pregnancy and lactation are energetically expensive [20]. As a result, the highly variable rainfall patterns in South Texas are strongly correlated with fawn recruitment [21]. These rainfall and fawn recruitment patterns result in frequent density-independent population dynamics [22–25]. Adult survival must be high to maintain deer populations in this semi-arid region. For instance, up to 40% of adults are ≥6 years old in unharvested populations [26]. In deer populations afflicted with CWD, prevalence increases with deer age [27,28]. Thus, introduction of CWD into the semi-arid regions of Texas may reduce the survival rates of adult deer in the population and have long-lasting effects on population size and structure.

South Texas has a unique association with hunting and management of white-tailed deer due to their ecological, economic, and cultural importance [19,29,30]. Because 97% of Texas is privately-owned, deer management in South Texas has become a viable business model (~$650 million US in South Texas annually [31]). Hunters are willing to pay for the privilege to hunt deer on private land; land-owners are willing to incorporate deer and habitat management strategies into their land management to increase revenue and hunter satisfaction. Furthermore, land prices reflect the potential of property to support wildlife recreation, especially deer hunting, because many people purchase land to have a place to hunt [32,33]. The relationship between landowners and hunters also benefits rural economies [34]. Because semi-arid rangelands are not consistently productive, deer managers often incorporate one or more intensive deer management techniques. Supplemental feed is commonly employed to increase fawn production, population size, and antler size [35,36]. Privately funded deer translocations are used to augment areas with low deer populations [37]. In management programs where production of large-antlered males is a goal [38], there are programs to control breeding of wild deer and to introduce captive-bred deer in wild deer populations [39].

The population-level effects of CWD are not well understood [40]. Population models have been used to project the effects of CWD on deer population dynamics [12,41–44]. Forecasting population trends is important because an additional source of mortality in a variable environment could alter the dynamics of compensatory and additive mortality [45]. Most models forecast a decline in cervid populations exposed to CWD, but the magnitude and severity of such declines have varied [43]. Some differences in model projections may be a function of parameters used for disease transmission rates. For instance, probabilities of disease transmission between susceptible and infected individuals may be modeled based on animal density (density-dependent) or not (frequency-dependent), or be based on age- and sex-specific prevalence rates [41,45]. However, transmission rates based on animal contact rates may be of secondary importance given that CWD may be transmitted through environmental sources, where prions may remain viable for years [12,46].

Most models of CWD effects on cervid population trends have been based on data from temperate climates, which typically facilitate higher and more consistent fawn recruitment [12,42]. Herein, we use empirical data collected from field studies in South Texas to model hypothetical outcomes of a deer population exposed to CWD in a semi-arid environment. We expected deer populations in our models to decline after the addition of CWD.

## Materials and Methods

### Study Area

We used survey and harvest data collected over a 20-year period on a subset of the King Ranch in Brooks, Kleberg, and Kenedy counties (27°31.164 N, 97°55.149 W) within the South Texas region to parametrize our population models. Deer were harvested conservatively; 1–4% of the total estimated population were harvested annually. Although supplemental nutrition can increase deer productivity in semi-arid environments [22,47], we did not use data from lands that used supplemental feed [35] because the practice would probably be banned or discontinued if CWD became established in the region. This is because provision of supplemental nutrition congregates deer, and likely would increase rates of disease transmission [48,49]. Field data were collected on four privately owned tracts that totaled 55,505 ha (range = 6,106–22,530 ha) in the Rio Grande Plains ecoregion [50,51]. The climate is semi-arid and subtropical with prolonged and frequent periods of drought. The vegetation community was dominated by Tamaulipan thornscrub [51]. Major land uses were cattle grazing and fee-lease hunting, with some dry-land agriculture and mineral exploration.

### Data Collection

We used a 20-year dataset of fawn:doe ratios and population counts collected from helicopter surveys [52] during September 1996–2015. Each year, a 2-seat (1996–1999) or a 4-seat (2000–2015) helicopter was used to survey each site via fixed-width transects (average annual total transect length = 928 km, range = 634–1,159 km). We derived an estimate of total population size by assuming a mean sighting probability of 0.3 to correct for visibility bias [53,54].

### Parameter Validation Model

**Fig 1:**
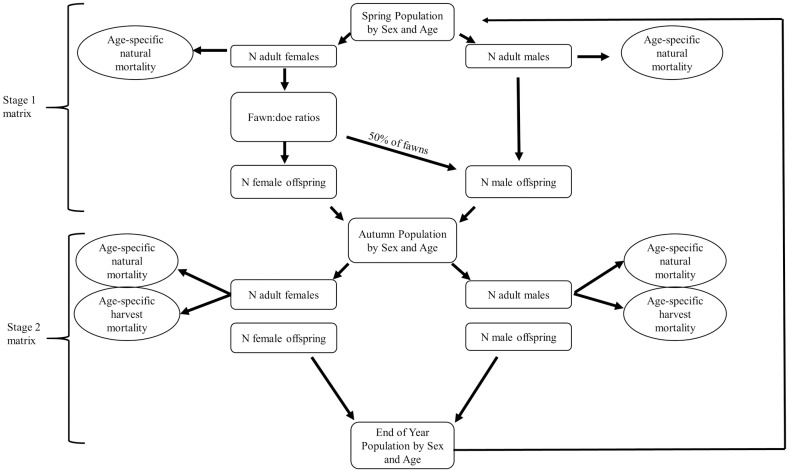
Schematic representing the white-tailed deer life cycle used in the parameter validation model.

We developed a multi-stage deterministic matrix model [55] that represented the life-cycle of white-tailed deer (Fig 1). The matrix incorporated age (fawn to ≥6.5 years old), sex, and sex- and age-specific survival rates. We used ≥6.5 years as our oldest age class because ageing via tooth-wear and replacement allows for relatively reliable separation between ≤5.5 and ≥6.5 years old [56]. Because the area surveyed increased during years 1996 to 1997, our initial population size for the first year in our parameter validation model was based on deer counts during 1997. Age of deer, other than fawns, cannot be reliably determined from helicopter surveys; thus, we constructed the initial population age structure. We assumed 40% of adult deer observed during the 1997 helicopter survey were ≥6.5 years old. This proportion of adult deer was based on deer captured as encountered with helicopter and captured with a net gun [57] on other properties in this region that were under similar deer management (e.g., no supplemental feeding and minimal harvest) [26]. For the remainder of deer observed in 1997 that were to be allocated towards the 1.5 to 5.5 year old age classes, we assumed a gradual decline in number of deer for successive age-classes because annual survival rates are <1 (Table 1).

**Table 1:** Initial population and age structure for the first year of the population model. Population size and sex ratio were derived from helicopter surveys and age structure was estimated based on field studies in South Texas.

Sex	Age	N deer	%
Male	1.5	712	15.8
	2.5	592	13.1
	3.5	522	11.6
	4.5	462	10.3
	5.5	414	9.2
	≥6.5	1801	40.0
Female	1.5	1362	14.6
	2.5	1237	13.2
	3.5	1112	11.9
	4.5	992	10.6
	5.5	913	9.8
	≥6.5	3744	40.0
Total		13863	

Because variable rainfall and fawn recruitment rates influence population structure, we input fawn:doe ratios in the order of year observed during our model validation efforts. Fecundity, defined as the fawn:doe ratio, was equal among all female age classes except for fawns (none). Although there is evidence that older females are more productive than younger females [58], we used the empirical fawn:doe ratios in the population as our measure of fawn recruitment.

Harvest occurred in the reference population and the number of harvested deer was known. We calculated the proportion of males and females that were harvested based on the annual deer counts corrected for visibility bias. The proportion of deer harvested, in order of year observed, was used to remove deer from the population following the birth pulse (Fig 1). The distribution of harvest was proportional to proportion of deer within each age class except fawns (none).

Sex- and age-specific survival rates were obtained from field studies in South Texas (Table 2). Survival rates were randomly drawn for each modeled year from a uniform distribution containing the range of reported survival rates. After running the parameter validation model, we slightly decreased survival rate of ≥6.5 year old deer about 10% relative to the average 2.5–5.5 year old survival rates until trends in the modeled data approximated the observed data. We focused on the survival rates of deer ≥6.5 years old 1) to minimize the accumulation of deer in the last age class, and 2) because there was some uncertainty about natural survival rates of deer ≥6.5 years old relative to younger deer, given the conservative harvest rates and lack of survival data.

**Table 2:** Reported field study values, parameter values, and references used to construct a population model that was compared with observed trends from deer helicopter surveys in South Texas.

Parameter	Field Study Values	Modeled Value	Reference
Female Survival: 1.5 years old	0.74 and 0.85	Annual random number selected from uniform distribution ranging between 0.74 and 0.85	[22,24]
Female Survival: 2.5–5.5 years old	0.85 and 0.93	Annual random number selected from uniform distribution ranging between 0.85 and 0.93	[22,24]
Female Survival: ≥6.5 years old	Unknown; assumed lower than average of 2.5–5.5 year old females	0.83	[22,24]
Male Survival: 1.5 years old	0.74, 0.80, and 0.85	Annual random number selected from uniform distribution ranging between 0.74 and 0.85	[22,24,59]
Male Survival: 2.5–5.5 years old	0.76, 0.78, 0.82, 0.88, and 0.92	Annual random number selected from uniform distribution ranging between 0.76 and 0.92	[22,24,60]
Male Survival: ≥6.5 years old	Unknown; assumed lower than average of 2.5–5.5 year old males	0.75	[22,24,60]
Recruitment rate	0.32, 0.49, 0.29, 0.57, 0.45, 0.22, 0.32, 0.37, 0.42, 0.14, 0.39, 0.35, 0.21, 0.14, 0.44, 0.36, 0.22, 0.22, 0.43, 0.67	Fawn:doe ratios during 1996–2015, in order of year observed	Empirical data
Fawn sex ratio	0.5	0.5	[61]
Harvest rate	1–7% of adult males and 1–6% of adult females	Proportion of annual count of adult males and females that were harvested, in order of year observed	Empirical data

Before conducting simulations, we ensured that our models produced realistic results by comparing trends between observed and modeled population trajectories. We compared model output to both annual estimates of population size and a 3-year moving average to smooth out variation in the year-to-year estimates [53,54].

### Results of Parameter Validation Model

**Fig 2:**
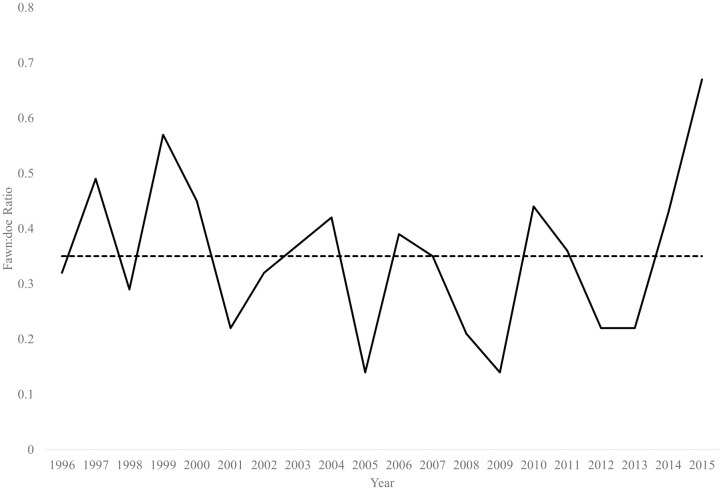
Fawn:doe ratios observed during helicopter surveys in South Texas, 1996–2015. Horizontal line indicates mean fawn:doe ratio.

**Fig 3:**
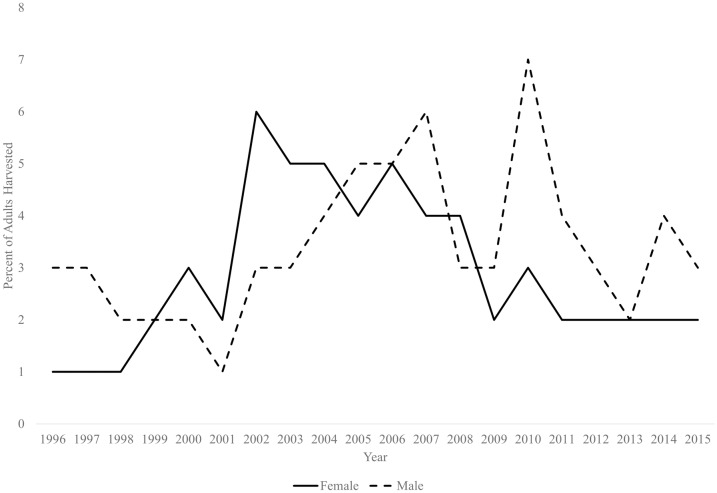
Percent of adult males and females harvested annually. Percent of deer harvested was based on number of adult males and females observed during September helicopter surveys in South Texas.

**Fig 4:**
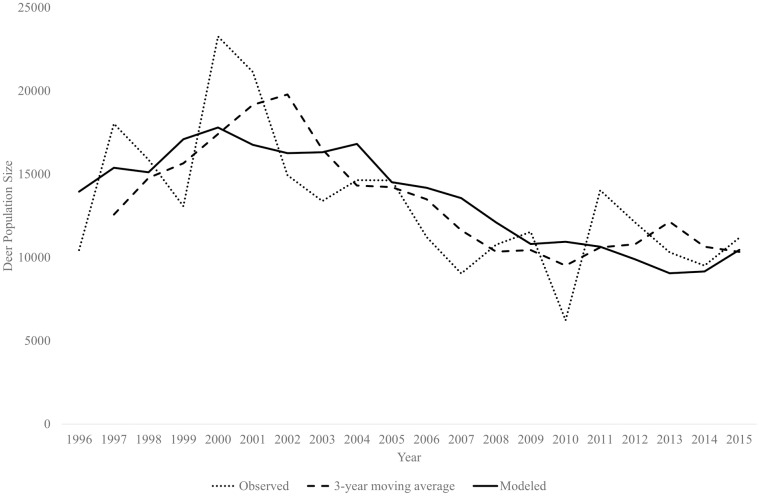
Comparison between observed, 3-year moving average, and modeled deer population size trends. White-tailed deer were counted via helicopter surveys in South Texas during 1996–2015 and compared well with output of parameter validation model (solid line).

During 1996–2015, observed fawn:doe ratios were highly variable (mean = 0.35, var = 0.02, range = 0.14–0.67, Fig 2). Annual harvest ranged from 1–6% and 1–7% of estimated adult female and male populations, respectively (Fig 3). The reference population experienced a decline after 2001, which likely reflected a succession of drought years that roughly aligns with the period of low fawn:doe ratios during 2005–2014. We produced a 20-year population trend that closely resembled trends in both observed annual deer counts and the 3-year moving average (Fig 4).

### Simulations of CWD

**Fig 5:**
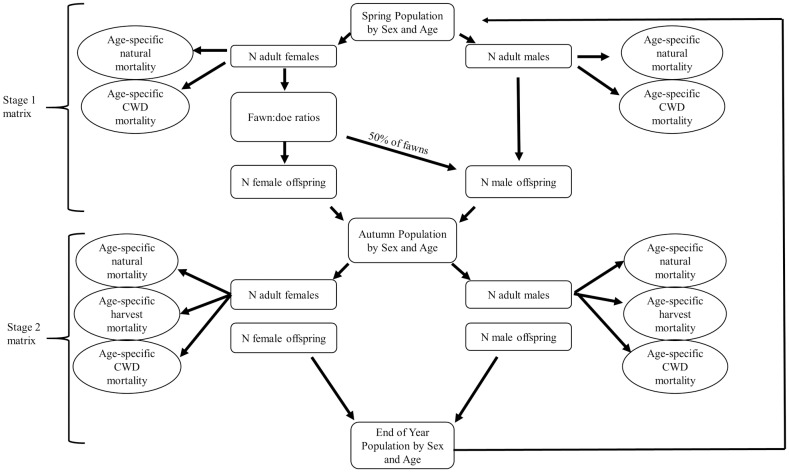
Schematic representing the white-tailed deer life cycle used in CWD simulations.

The simulation approaches had the same structure as the multi-stage deterministic matrix model that we used in the parameter validation model with the exception of an additional matrix to track the CWD-positive deer (Fig 5, Table 2). We simulated 4 25-year deer population scenarios: no CWD with no harvest, CWD with no harvest, CWD with male harvest, and CWD with harvest of both sexes. For simulations without deer harvest, we simply set harvest to zero. In simulations with harvests, harvest was entered as a constant in each model but we incrementally increased harvest (1%, 2%, etc. of adult deer removed from the population) for each additional model until the population declined at year 25. This approach was used to determine the maximum constant annual harvest rate a CWD-positive population could sustain without declining.

For the CWD parameters, we used age- and sex-specific prevalence rates from white-tailed deer in Wisconsin ([27], Table 3). The age- and sex-specific prevalence rates were weighted to reflect differential risk in terms of becoming infected. For instance, given 1,000 deer in a population with a 1% prevalence, 10 deer would be infected. Allocating the 10 infections towards the weighted sex- and age-specific prevalence rates (Table 3), 1.37 4.5-year old males would be infected (0.137 * 10 infected deer). Then in year 2, the number of new infections, after accounting for the infected deer present in the population, would be allocated again towards the appropriate sex- and age-classes. Mortalities due to CWD were assumed to be additive to natural mortality. We have no evidence that mortality events due to CWD in semi-arid environments would be compensatory [62] because deer population dynamics typically are density-independent and influenced more by highly erratic environmental conditions and subsequent changes in forage resources than by density [22–25]. Many life processes are negatively influenced when a population exhibits density-dependent population dynamics [63]; however, large-scale, longitudinal experimental studies in South Texas indicate adult body mass was the only life process influenced in a 4-fold difference in deer density [22,24]. Further, fawn recruitment in the same experimental study was not influenced by deer density [58].

**Table 3:** Initial population and age structure for the first year of the simulations. Sex- and age-specific numbers were derived from median values generated in year 7 of simulations based on South Texas data without CWD and without harvest.

Sex	Age	N deer	%
Male	1.5	1263	21.3
	2.5	1064	17.9
	3.5	877	14.7
	4.5	748	12.6
	5.5	641	10.8
	≥6.5	1333	22.4
Female	1.5	1261	15.3
	2.5	1122	13.6
	3.5	977	11.8
	4.5	879	10.6
	5.5	794	9.6
	≥6.5	3208	48.9
Total		14167	

Chronic wasting disease is a slow-spreading disease that increases in prevalence over time [64]; therefore, we opted to model CWD prevalence growth rates derived from 3 afflicted areas. West Virginia, Wisconsin, and Wyoming exhibited 0.26%, 0.83%, and 2.3% annual increases in CWD prevalence, respectively [12,65,66]. We classify these 3 growth rates as slow, medium, and rapid, respectively. The growth rate of CWD prevalence when first introduced into a system is unknown because CWD is not typically detected in an area until prevalence is ≥1% [42]. Thus, we elected to start at a prevalence at 1% for each of the 3 CWD growth rates which reflects the scenario where CWD is discovered in a wild deer population in South Texas and presumably un-eradicable [44]. For each year in our simulations, prevalence started at 1% of the population then increased annually for 25 years in increments according to of the particular prevalence growth rate modeled.

Prevalence does not specify mortality rates, so we assumed that deer expired 1 to 3 years after contracting CWD [1]. A randomly chosen value of 33%, 50%, or 100% mortality rate was applied towards the subset of deer infected with CWD. Mortality from CWD was modeled to occur year-round, with no seasonal variation.

The social nature of white-tailed deer results in CWD transmission rates that are not entirely density-dependent [67–69]; thus, we did not incorporate a density-dependent disease transmission rate in the model. Further, we did not incorporate spatial components in our model because relative to the upper Midwest and Intermountain West where there is a high diversity in land-cover (and deer density), South Texas is relatively homogenous with large swaths of Tamaulipan thornscrub where deer density is unlikely to vary greatly.

For the initial population sizes in the simulations, we opted to use the median values of sex- and age-specific population sizes generated by year 7 in the simulations without CWD and without harvest (Table 3). This was done because the number of 1.5 year old males in our original initial sex- and age-specific numbers (Table 1) was ~50% lower than the number generated by our simulations in year 2. The abrupt increase in 1.5 year old deer carried through years 2 to 6 and resulted in distorted proportions which influenced the ability to produce appropriate annual-based statistics such as changes in population size and prevalence. Sex- and age-specific survival rates were identical to the parameter validation model (Table 2). Instead of using the observed time series of fawn:doe ratios, we used random draws from the ratios during 1996–2015. The inclusion of random draws from observed fawn:doe ratios allows the incorporation of environmental stochasticity, an important consideration in the semi-arid environment. To further evaluate the influence of variable environment on population-level effects of CWD, we also modeled population trajectories based on fawn:doe ratios from 3 areas afflicted with CWD in Wyoming and Wisconsin (Table 4). The South Converse mule deer herd in Wyoming had an average of 0.54 fawns per doe (range = 0.43–0.73 [70]) and the Laramie Mountain mule deer herd had an average of 0.63 (range = 0.51–0.81 [71]). We randomly selected 6 Wisconsin counties where CWD was present and the average fawn:doe ratio was 0.84 (range = 0.62–1.00 [72]). For each scenario with the 4 CWD growth rates (none, slow, medium, and rapid) in conjunction with male and female harvests (none, 1%, 2%, etc.), we simulated 25-year population trajectories with 1,000 iterations. All statistical operations and modeling were done in the R programming environment [73] (S1 File).

**Table 4:** Parameter values used to simulate CWD effects on deer population dynamics in South Texas.

Parameter	Value	Reference
Recruitment rate: Semi-arid	Randomly selected from empirical fawn:doe ratios observed during 1996–2015	Empirical data
Recruitment rate: Temperate	0.44,0.40,0.72,0.49,0.46,0.73 (Converse, WY)	[70–72]
	0.66,0.62,0.51,0.59,0.61,0.81 (Laramie, WY)	
	1.00,0.76,0.87,0.90,0.88,0.62 (Wisconsin)	
Population prevalence growth	Started at 1% and increased 0.26%, 0.83%, or 2.3% annually for 25 years	[12,65,66]
Female CWD risk: 1.5—≥3.5 year old	2.3, 3.8, and 6.1%, respectively	[27]
Male CWD risk: 1.5—≥6.5 year old	2.3, 7.6, 9.9, 13.7, 16.8, and 19.1%, respectively	[27]
CWD mortality rate	Randomly selected 33%, 50%, or 100% mortality rate applied towards subset of infected males and females	[1]
Harvest	Constant within each model and increased at 1% increments for each additional model until population declined	

### Results of CWD Simulations

**Fig 6:**
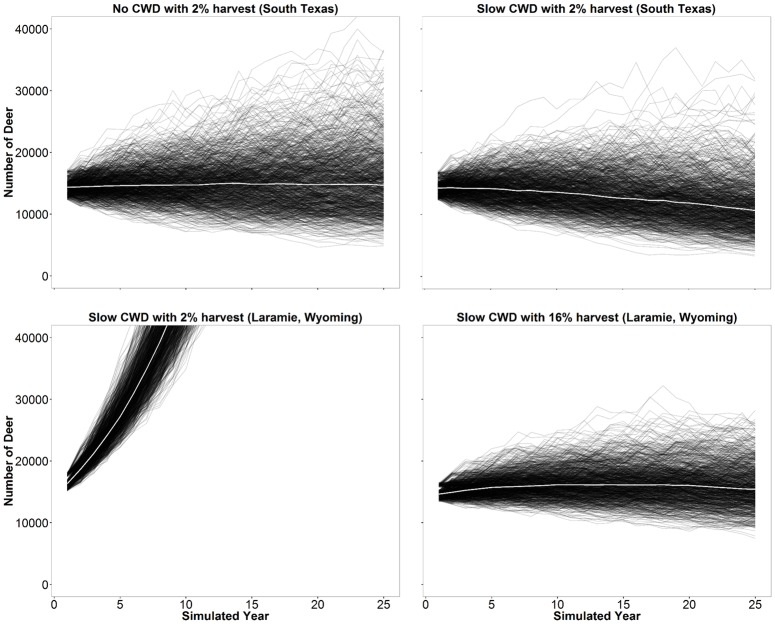
Plots of simulated white-tailed deer population trajectories. One thousand 25-year simulations were run to predict future populations without CWD and 2% harvest (top left) and with CWD and without harvest (top right) with fawn:doe ratios from South Texas, with CWD and 2% harvest (bottom left) and 16% harvest (bottom right) with fawn:doe ratios from Laramie, Wyoming. Slow CWD was modeled to increase 0.26% annually. White line indicates median of the 1,000 simulated projections.

**Fig 7:**
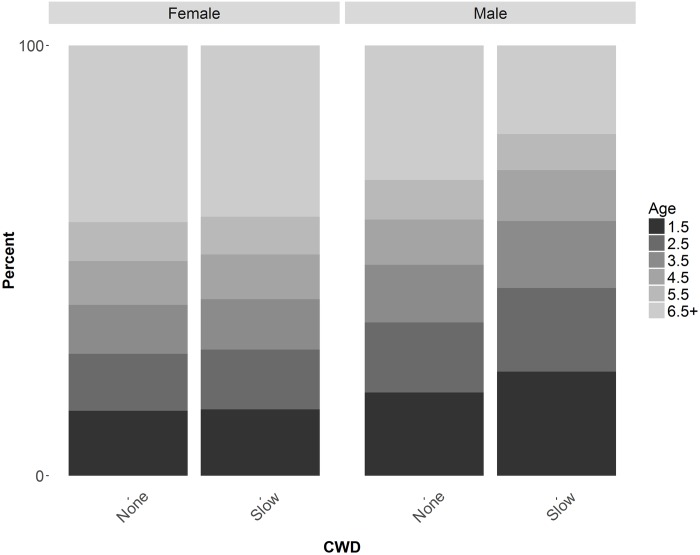
Age structure of male and female population after 25-year simulations with and without CWD. Slow CWD started at 1% prevalence and increased 0.26% annually.

**Fig 8:**
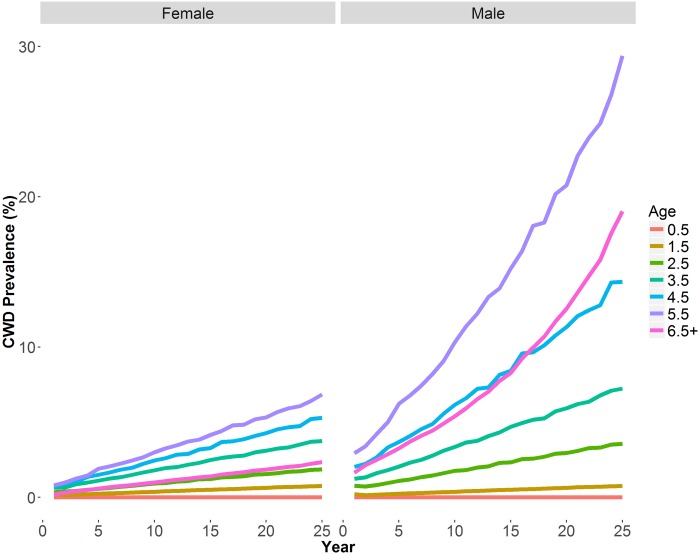
Sex- and age-specific prevalence at the end of simulated years in the slow CWD increase model. Prevalence is the percent of the median number of infected sex- and age-specific deer in the population at the end of each simulated year. Slow CWD started at 1% prevalence and increased annually by 0.26%.

Simulations without CWD and without harvest in the model indicated an increasing population trajectory that averaged an annual growth of 1.43% resulting in a net 36% increase after 25 years (Fig 6). Inclusion of CWD in the model without harvest resulted in 0.41, -1.72, and -10.33% annual rates of change in deer populations with slow, medium, and rapid CWD growth rates, respectively. Harvest of 1% of females resulted in a population decline in the slow CWD model but when harvest was limited to males, populations did not decline. Relative to simulations without CWD, the introduction of CWD produced nearly identical female age structures; however, there was a shift in male age structures (Fig 7). Relative to the model without CWD, the different age-specific CWD prevalence rates resulted in an increase of proportion of young males while proportion of ≥5.5 year old males declined. Percent of infected deer in the population at end of modeled years followed expected trends relative to inputted CWD parameters (Table 4) except the ≥6.5 year old male age class was lower than expected (Fig 8).

**Fig 9:**
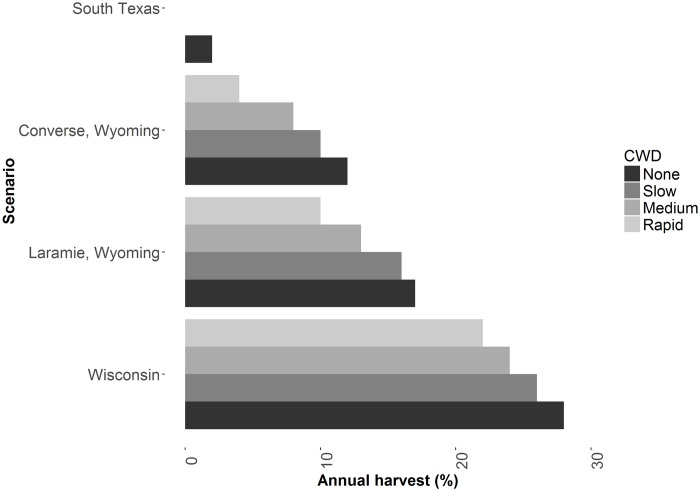
Maximum annual harvest of adult male and female deer with slow, medium, and rapid CWD prevalence growth rates. CWD increased annually at rates of 0.26% (slow), 0.83% (medium) or 2.3% (rapid). Absent bars indicate scenarios when harvest of both sexes caused a population decline.

Substituting our empirical South Texas fawn:doe ratios with observed fawn:doe ratios from temperate areas afflicted with CWD resulted in large annual population increases in all slow and medium scenarios. Populations stabilized when ~10%, ~16%, and ~26% of the deer were harvested annually in slow CWD growth models based on fawn:doe ratios from South Converse, WY, Laramie Mountains, WY, and Wisconsin, respectively (Fig 9).

## Discussion

Our simulations suggest that additional adult mortality due to CWD will result in reduced or negative rates of population growth for populations of white-tailed deer in semi-arid environments. In regions with variable rainfall, adult survival must be high to overcome variable fawn recruitment [24]. The additive mortality of CWD in conjunction with higher prevalence rates in adult deer resulted in a reduced population size, even in the absence of hunter harvest of deer. Whereas CWD-afflicted deer populations elsewhere have been able to persist for over 30 years [42,74], the higher mean recruitment in conjunction with low prevalence rates in younger deer [12,28] likely enables population sustainability. Model projections based on fawn:doe ratios from temperate environments instead of semi-arid environments support the idea that high deer productivity increases likelihood of population sustainability. In our South Texas scenario with no harvest and slow CWD increase, a negative rate of population growth did not occur; therefore, deer populations in semi-arid regions may be able to persist after CWD introduction in the absence of harvest. For simplicity, we did not consider age-specific fecundity in our models. However, deer productivity in semi-arid environments is strongly influenced by age of the dam [58]. Therefore a decline in the age structure of females due to increased CWD prevalence [27,28] would likely result in a more rapid decline in our population simulations. The increased rate of population decline would be attributed to the reduced number of older, more productive, females available to produce offspring. Thus, young females (≤2.5 years old) would be the critical factor driving population sustainability in CWD-afflicted areas. However, there may not be sufficient healthy, and less productive, young females available to produce offspring in semi-arid environments because the lower and more variable fawn:doe ratio (0.35, SE = 0.03) results in a variable young female age structure [26].

CWD mortality was modeled to be additive because of the density-independent nature of deer dynamics in South Texas (22,24). It is possible that CWD mortality would be compensatory because of predation [75], deer-vehicle collisions [76], and harvest [77]. Mountain lions (*Puma concolor*) and coyotes (*Canis latrans*) exist in South Texas and may selectively prey on infected deer [74,75]; however, mountain lion density in South Texas is low (0.59–0.75 per 100 km^2^ [78]). Coyotes and bobcats occasionally kill adult deer [79] but primarily prey on fawns [80,81], which are not considered important to CWD dynamics [82]. Deer-vehicle collisions are not as frequent in South Texas compared to other states because of the relatively low road density; thus, it is unlikely deer-vehicle collisions would have an influence on CWD mortality. Deer mortality events due to harvest could be compensatory because both CWD prevalence and probability of being harvested increases with age [27]. However, CWD presumably does not discriminate by antler characteristics whereas hunters generally select for large-antlered mature males. Overall, it would be expected for CWD mortality to transition from compensatory to additive as prevalence rate increases because survival rates would be reduced [83,84]. The point in time when CWD mortality becomes additive is unknown but in deer populations that rely on high survivorship of adult deer, the transition from compensatory to additive is likely earlier relative to areas with higher recruitment typical of temperate environments.

If our hypothetical CWD-afflicted population was able to sustain itself, perhaps during a period of favorable rainfall and increased fawn:doe ratios [18,85,86], the higher prevalence rates in mature males is of concern. Our CWD simulations forecasted a male age structure with fewer mature (≥5.5 year old) males (Fig 7). Male harvest in south Texas is generally skewed towards mature males [60] and the change in CWD-modeled male age structures will likely be more pronounced because we modeled harvest to be equal among male age classes. Further, prevalence rates of ≥6.5 year old males at the end of simulated years were lower than expected (Fig 8) which suggest that CWD mortality rates would have a disproportionate effect on old (≥6.5 year old) males [27]. Culturally and economically, harvest of mature males is a critical component of hunting in South Texas [38]. With fewer mature males in CWD-afflicted populations, deer hunting may not be economically viable for privately owned ranches. Ranches may transition to alternative sources of income, such as agriculture or development which may not have the same ecosystem benefits as wildlife-cattle management programs on native rangelands [87–89].

Declines in our simulated CWD populations with only 1% female harvest suggest that a slight increase in additive mortality rates would be unsustainable for deer populations in semi-arid regions. This finding is not unexpected because deer managers have long known that harvest rates in native rangelands, where deer population density-independence occurs frequently, need to be conservative to prevent population declines due to the additive nature of harvest [24,90]. The additive mortality via harvest suggests that increased harvest in an attempt to suppress CWD [3,45,91,92], depending on the prevalence rate [44], may be a viable management strategy. However, the social nature of white-tailed deer (i.e., bachelor male groups and female family groups [93,94]) does not result in prevalence rates that are positively correlated with deer density [67,68]. Additionally, the persistence of prions in the environment complicates disease management strategies. This may be more so in semi-arid environments, where the availability of drinking water plays a critical part in habitat use during dry years [95]. One symptom of CWD is increased thirst [96]; thus, frequent visits to water catchments, troughs, stock tanks, and ephemeral pools [97] may increase disease transmission rates and congregate prion deposition [98]. Some natural water sources may be associated with impermeable clay soil, which may increase viability of CWD prions [12,13,99].

We acknowledge that our relatively simplistic population model contains both process variation (temporal, individual, and demographic variation) and sampling variation (variation in measured parameters) which could lead to bias in population projections [100,101]. However, we believe the impact of both types of variation on our models is relatively low. For instance, density dependence influences many life processes [25,63]. Because density dependence is rarely observed in south Texas [22,24], our model is unlikely to need density-dependent changes in vital rates as a result of a decrease in deer density. Another important component of population models is environmental stochasticity. In South Texas, environmental stochasticity is represented by variable rainfall which influences fawn production (i.e., fawn:doe ratios) [21], which was an integral part of our model. Further, it is well documented that fawn recruitment rates are the demographic parameter influencing deer population growth because adult deer have high natural survival rates [102]. Therefore, by controlling our models with known and critical region-specific parameters [103] and increasing prevalence rates at a rate observed in three CWD-afflicted areas [12,65,66], we feel our model is a fair demonstration of what could happen if CWD emerged in South Texas. The first CWD positive white-tailed deer identified in Texas was found in a captive deer breeder facility. The second CWD case occurred at a different captive facility was a result of an epidemiological investigation of deer purchased from the index facility. As of July 2016, 25 white-tailed deer in or originating from captive facilities have been confirmed positive for CWD in Texas [104]. Within Texas, there are >1,300 breeder facilities that house >110,000 white-tailed deer [105]. There is a risk of fence-line transmission between captive and free-ranging deer [106,107] but perhaps the bigger risk lies in the practice of deer translocation. In 2014, 27,684 deer were translocated from captive deer facilities to other captive deer facilities, high-fenced (surrounded by 2.5-m high woven-wire fence) properties, and low-fenced (1.25-m) properties [105]. Releasing a deer unknowingly infected with CWD may result in disease transmission to a native deer population via horizontal transmission or deposition of prions into a new environment.

Chronic wasting disease is a slow-spreading disease that may take years or decades to result in detectable prevalence rates [1]. Modeling is frequently used to project CWD consequences to cervid populations because field experiments are impractical [12,43,67]. Our simulations suggest that introduction of CWD into deer populations with low fawn production and that frequently exhibit density independence would have a significant impact on population size and age structure. Management efforts to enhance deer populations in this region in the event of CWD introduction would likely be difficult or infeasible. For instance, using supplemental nutrition may increase deer productivity to combat deleterious effects of drought years and CWD mortality; however, presence of feed stations would likely increase disease transmission rates [35,48,49]. Transplanting deer from CWD-free populations may be a feasible option; however, the prolonged existence of CWD prions in the environment will likely result in disease persistence. Therefore, prevention of CWD introduction into variable environments such as South Texas is a critical strategy to ensure deer continue to exist as a renewable natural resource.

## Supporting Information

S1 File R codes for population simulation of chronic wasting disease in white-tailed deer. (TXT)
